# Developing the Creative Abilities of Children with Learning Differences in Rural, Low‐Resource Settings in Africa: A Case Series Study

**DOI:** 10.1002/brb3.71287

**Published:** 2026-02-27

**Authors:** Naomi Beth Conolly, Munira Hoosain, Doranne McDonald, Janke van der Walt, Nicola Ann Plastow

**Affiliations:** ^1^ Division of Occupational Therapy Faculty of Medicine and Health Sciences, Stellenbosch University Cape Town Western Cape South Africa; ^2^ Neurodiversity Foundation Cape Town Western Cape South Africa

**Keywords:** community‐based intervention, creative ability, learning differences, occupational therapy

## Abstract

**Introduction:**

Creative ability is an important outcome of developmental support programs, as it helps to lay the foundation for better long‐term participation and well‐being. Children with learning differences risk underdeveloping this potential, especially those in rural, low‐resource African settings.

**Aim and Methods:**

This study aimed to determine the potential outcomes of *Create2Grow*, a new community‐based occupational therapy visual arts group intervention for children aged 8 to 12 years with mild to moderate neurodevelopmental disorders, using a case series research design with eight children recruited via convenience sampling.

**Results:**

The intervention had a large clinical effect on participants’ caregiver‐rated Canadian Occupational Performance Measure scores (*d* = 1.5) and clinician‐rated Creative Participation Assessment scores (*d* = 1.76) from pre‐ to post‐intervention. *Create2Grow* was also rated as highly in demand, acceptable, and practical for the target group. Low study attrition rates showed that the intervention could be effectively implemented at a local school within the community.

**Conclusion:**

*Create2Grow* is a promising solution to promoting the creative ability of children with learning differences in rural, low‐resource settings in Africa.

## Graphical abstract

*Create2Grow*, an eight‐week community‐based occupational therapy visual art group intervention, demonstrated large improvements in occupational performance and creative participation among children with mild to moderate learning differences and was feasible to implement in a rural, low‐resource African setting.

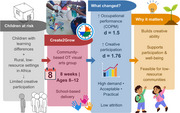

## 1 Introduction

Creative ability, closely linked to adaptability and resilience, is increasingly recognized as a protective factor that facilitates enduring developmental success (Macpherson et al. 2016; Zarobe and Bungay 2017). This suggests that when children actively participate in purposeful, voluntary, and creative occupations (daily activities), they can achieve higher levels of self‐efficacy, school readiness, resilience, social integration, executive functioning, and adaptive coping skills (Casteleijn et al. 2019; Conolly et al. 2025; de Witt 2014; Ginsburg 2007; Lester and Russell 2008).

However, children with learning differences often experience challenges with participating in developmentally relevant roles (such as a player, learner, and family member), particularly when their ways of thinking and being are not valued or supported (Kuzmanov 2025). Therefore, there is a need for scalable and innovative solutions that enable access to neuro‐inclusive practices to help children with learning differences realize their developmental potential.

Children with learning differences in rural, low‐resource settings in Africa face greater barriers to developing their creative ability, as their learning and developmental challenges often remain undetected and unsupported (Walton and Rusznyak 2019). This lack of access is largely due to resource constraints, fiscal mismanagement, and underdeveloped infrastructure (Maphumulo and Bhengu 2019; Nash et al. 2024; Patel 2025; Townsend and Wilcock 2004; van Zyl et al. 2021) and tends to worsen long‐term symptomology. This, in turn, reinforces socioeconomic issues such as poverty, crime, and unemployment (Kleintjes et al. 2022).

To change this current trajectory, healthcare professionals need to empower local, community‐based change agents to promote the early identification of neurodevelopmental challenges and referrals to accessible intervention services. To achieve this, layman's language needs to be used, given the educational inequalities common in rural, low‐resource settings (van Zyl et al. 2021). Therefore, the term “learning differences” can be used to describe the variety of early‐onset and chronic neurodevelopmental conditions that hinder a child's socio‐emotional, academic, cognitive, communication, and life skills development (Bell 2023; Okyere et al. 2019). Community members, teachers, and caregivers who can observe these differences in functioning that ultimately affect learning can then be mobilized to flag and refer neurodivergent children for timely intervention (Conolly et al. 2025).

International research on the prevalence of neurodivergent conditions reveals that as many as 15% to 20% of people fall under the neurodiversity umbrella (Bell 2023; Kuzmanov 2025). However, in South Africa, like in many other rural, low‐resource settings in Africa, issues such as stunted growth, adverse childhood experiences, unsupported neurodivergence in parents, and a lack of early childhood development, healthcare support, and accommodative education compound the negative, long‐term outcomes that children with learning differences face (Aafjes‐Van Doorn et al. 2020; Burger et al. 2020; Okyere et al. 2019). In the Western Cape region of South Africa, it is estimated that 64% of children in low‐resource areas have developmental delays in at least one domain, and 72% to 75% are exposed to abuse, neglect, and/or violence (Chademana et al. 2023; Tsunga et al. 2023). These statistics highlight that children with developmental and learning differences, intensified by traumatic exposures, represent a large portion of the population.

As a potential solution to the issue of resource constraints and undetected and unsupported learning differences in rural, low‐resource contexts, community‐based group interventions of a creative and developmental nature stand out as a growing evidence‐based approach (Conolly et al. 2025; Klasen and Crombag 2013; Sofija et al. 2023; Van Westrhenen et al. 2019). Such interventions have the benefit of being adaptable to different contexts and are well‐suited to enhancing the foundational skills required for individual development and participation in everyday life (Conolly et al. 2025). For example, when Van Westrhenen et al. facilitated a 10‐session psychotherapy creative arts group for 7 to 13 year old children with a history of trauma exposure or behavioral difficulties within a community context, the intervention resulted in significant decreases in hyperarousal and avoidance behaviors (*d* = 0.61 and 0.41, respectively) (2019).

Community‐based group therapy programs may present the most feasible method to ensure that more children benefit concurrently (Klasen and Crombag 2013). Developmental support interventions also tend to have a good return on investment, given better long‐term socio‐economic and health outcomes (Heckman 2015), thereby saving costs in the long run. However, to prioritize programs with the best chance of clinical effectiveness and feasible expansion, the preliminary quantitative and qualitative elements of novel interventions need to be well defined before more rigorous testing can be conducted (O'Cathain et al. 2019; Teresi et al. 2022).

Therefore, this study aimed to determine the potential outcomes of eight weekly 45‐minute occupational therapy visual art groups for eight children (aged 8 to 12 years) with learning differences in a rural, low‐resource setting in the Western Cape, South Africa. The program focuses on the development of creative ability, as, according to the Vona du Toit Model of Creative Ability, motivation to act and exert maximum effort leads one closer to one's potential, thereby creating opportunities for individual development or growth (de Witt 2014).

## 2 Methodology

Quantitative, experimental research in the form of a case series design was utilized for this study. A positivist approach asserts that reality can be objectively assessed through deductive reasoning and can be measured in a manner that can bear statistical significance (Terre‐Blanche and Durrheim 2006). Therefore, quantifiable pre‐ and post‐intervention measures were used with a series of individuals receiving the same intervention, without a comparison or control group, to determine the preliminary outcomes of the intervention (Dekkers et al. 2012).

This prospective research evaluates the potential effectiveness of the intervention, its suitability for a rural, low‐resource setting, and the feasibility of its methods and procedures for future larger‐scale studies (Billingham et al. 2013; Eldridge et al. 2016; Teresi et al. 2022). The Standard Protocol Items: Recommendations for Interventional Trials (SPIRIT 2013 Statement) were used to guide the development of the research design (SPIRIT Group 2013).

### 2.1 Recruitment

Recruitment was done in collaboration with the Neurodiversity Foundation (NDF), a non‐profit organization offering developmental support services in the Breede Valley of the Western Cape. This rural, low‐resource setting is primarily a table‐grape farming community with a high prevalence of developmental challenges and social issues (Truter et al. 2025). Once recruitment flyers were distributed in the community and various stakeholders assisted with the referral of potential participants, screening was done according to the criteria in Table 1.

**TABLE 1:** Inclusion and exclusion criteria.

Inclusion criteria	Exclusion criteria
Children between the ages of 8 and 12. Children who met the DSM‐5 criteria for a neurodevelopmental disorder diagnosis with a mild/moderate presentation of ASD, ADHD, ID, SLD and/or a communication or motor disorder. Conditions associated with medical, genetic, and/ or environmental factors were accepted, for example, ID related to fetal alcohol syndrome or Down syndrome. No formal diagnosis (beyond the initial DSM‐5 screening) was required. Residents from De Doorns in the Breede Valley Municipality, Western Cape, South Africa.	Children with physical disabilities or co‐morbidities such as cerebral palsy, spina bifida, muscular dystrophy, deafness, or blindness. Severe to profound presentations of neurodevelopmental disorders. Children who were receiving other therapy services.

**Abbreviations**: ADHD, attention‐deficit/hyperactivity disorder; DSM‐5, Diagnostic and Statistical Manual of Mental Disorders (fifth edition); ID, intellectual disability; SLD, specific learning disorders.

Convenience sampling was used, given resource constraints and educational disparities, as most caregivers were unaware of learning differences and required guidance and psychoeducation to identify learners who met the inclusion criteria. No more than eight participants were recruited to ensure that each learner received the appropriate amount of support during the groups, as there were only two intervention providers available for group facilitation. A larger sample size was not possible, given pragmatic reasons such as time, transport, and financial limitations.

The NDF's clinical psychologist (D. M.) screened eligible participants using the neurodevelopmental disorder criteria in the DSM‐5 until eight participants were identified. Adult consent and child assent forms were made available in participants’ home language, and translators were incorporated as needed to ensure that each participant's questions were fully answered and the voluntary nature of the study was understood.

### 2.2 Data Collection

An external assessor, an occupational therapist‐researcher (*J. vd. W*.), completed the COPM and COSA with the participants and their caregivers in individual sessions at pre‐ and post‐intervention. Informal Afrikaans translation guides, generated by a mother‐tongue speaker, were provided for the assessor for the facilitation of the COPM and COSA. These guides were not anticipated to have jeopardized the validity of the assessment, as both assessment manuals emphasize the need for client‐centered and conversational facilitation. The assessor also observed the first and last intervention sessions to score the participants on the observational assessment of the CPA, independent of the occupational therapist who facilitated the groups. Demographic data, informed by the PROGRESS‐Plus health equity criteria (Cochrane 2014), were collected by the principal investigator (*N. B. C*.) and clinical psychologist.

Within the COPM and COSA, the ability to participate in everyday tasks is defined as occupational performance (Law et al. 1990). The Canadian Model of Occupational Performance and Engagement (CMOP‐E) views occupational performance as the balance between a person's performance in three key domains: self‐care, productivity, and leisure—daily activities that a person wants, needs, or is expected to engage in (Law et al. 1990). Therefore, performance in these domains translates to one's active participation in a variety of developmentally appropriate and culturally relevant everyday activities. Similarly, in the Vona du Toit Model of Creative Ability (VdTMoCA), creative participation is regarded as “the process of being actively involved in activities and occupations concerned with everyday living relevant to the individual's level of development” (de Witt 2014). The ability to participate is influenced by one's creative ability, or preparedness to function freely and with originality at the maximum level of competence (de Witt 2014). Therefore, creative ability is the result of various physical, cognitive, and psycho‐social enablers that determine the quality and nature of participation. To measure changes in creative ability, three outcome measures were used.

### 2.3 The Canadian Occupational Performance Measure (COPM)

This patient‐rated outcome measure is widely used in occupational therapy clinical practice for people over the age of 8 years old (Ohno et al. 2021). However, in this particular study, it was used to determine the primary caregivers’ views of their children's quality of participation in activities they deemed important and culturally relevant. A semi‐structured interview enabled caregivers to identify five important occupational performance concerns relating to their children and rate them on a scale of 10 on the COPM‐performance and COPM‐satisfaction subscales (Law et al. 1990; Ohno et al. 2021). Total scores are calculated by adding the subscale scores separately and dividing the score by the number of problems identified. In the post‐intervention assessment, the caregiver scores each concern again according to performance and satisfaction. On the COPM, a two‐point improvement in performance and satisfaction scores from pre‐ t.o post‐test indicates a clinically significant change in overall occupational performance (Law et al. 2015).

### 2.4 The Creative Participation Assessment (CPA)

The CPA is a Vona du Toit Model of Creative Ability (VdT MoCA) model‐based outcome measure used to capture a clinician's observations regarding a person's level of creative ability across different tasks and situations. A person's activity and social participation are measured according to their levels of action and motivation, as plotted on the nine creative ability levels, which contain various descriptors. Within each level, a person is plotted within a ‘therapist‐direct,’ ‘patient‐directed,’ or ‘transitional’ phase based on the level of support required for activity participation (Casteleijn et al. 2019).

The measure analyzes criteria such as behavior, tool handling, task initiation, and ability to sustain participatory efforts to determine their level of creative ability. Intervention facilitators also used the CPA after every second or third session to monitor progress and tailor the intervention to present a just‐right challenge for each participant (Case‐Smith and Clifford O'Brien 2014). These results were not shared with the external assessor to prevent facilitator bias in the CPA scores.

### 2.5 The Child Occupational Self‐Assessment (COSA)

The self‐report assessment gives children the opportunity to rate their level of competence in performing different daily activities and evaluate the value they attach to these tasks according to a four‐point emoticon‐based Likert scale (Ohl et al. 2015). This assessment is appropriate for children between 8 and 13 years old and can also be used with younger children or those who have mild intellectual disabilities, should they have the necessary self‐reflection skills (Ohl et al. 2015). If participants had difficulty understanding a question, the assessor was able to rephrase it in simpler terms to foster comprehension, as child‐specific adaptation is encouraged in the COSA manual (Ohl et al. 2015).

The assessment includes 25 items and three follow‐up questions. The competence ratings are converted into a frequency table, which places the items on a scale of “big problems” (rating = 1) to “really good” (rating = 4). Everyday activities that are regarded as “not really important to me” or do not have competency ratings are excluded. The total of the competence values provides an “actual score,” and a percentage of the maximum possible score (POMP) is calculated as follows:

$$\begin{array}{ccc} & & \mathrm{Lowest}\;\mathrm{possible}\;\mathrm{score}=\\ & & \left(\mathrm{Number}\;\mathrm{of}\;\mathrm{competence}\;\mathrm{items}\;\mathrm{rated}\;\mathrm{as}\;\mathrm{important}\right)\;\times 1 \end{array}$$

$$\begin{array}{ccc} & & \mathrm{Highest}\;\mathrm{possible}\;\mathrm{score}=\\ & & \left(\mathrm{Number}\;\mathrm{of}\;\mathrm{competence}\;\mathrm{items}\;\mathrm{rated}\;\mathrm{as}\;\mathrm{important}\right)\times 4 \end{array}$$

$$POMP=[\left(``actual^{\prime \prime }-``lowest^{\prime \prime }\right)/\left(``highest^{\prime \prime }-``lowest^{\prime \prime }\right)]\times 100$$

This score indicates a participant's sense of competence for all items he/she felt were important. The POMP score is used to measure change from pre‐ to post‐intervention. A higher percentage indicates improvement.

### 2.6 Intervention

The eight participants attended weekly 45‐minute group sessions over eight weeks from September to October 2023. Groups were limited to eight sessions, given findings from a pre‐intervention systematic review indicating that longer art group interventions are not necessarily more effective than short‐term interventions using goal‐directed and evidence‐based methods (Conolly et al. 2025). The group met on the same weekday at the same time, directly after school, in the mid‐afternoon. The groups were facilitated by two clinicians: the principal investigator, an occupational therapist (*N. B. C.)*, and a clinical psychologist (*D. M.)*, with the inclusion of a translator‐facilitator to ensure that one participant could engage optimally in the groups. Therefore, with two group facilitators and eight group participants, the ratio of therapist to participant was 1:4.

A variety of occupational therapy models based on group therapy, developmental, and motivational theory were consulted in the development of *Create2Grow* (Casteleijn et al. 2019; Cole 2018; de Witt 2014; Kramer et al. 2010), alongside low‐to‐middle‐income country intervention research and psychosocial development theory (Chung 2018; Klasen and Crombag 2013; Ryan and Deci 2017). The AR^3^T Principles Framework, a guideline for art group interventions for children with learning differences, was also used to ensure that each session had a nurturing facilitator, opportunities for self‐ or co‐regulation, a set routine with predictable behavioral boundaries and consequences, and realistic materials and methods tailored to each individual's level of ability (Conolly et al. 2025). Sessions consisted of paper mâché, acrylic painting, tracing with wax crayons and paint‐washing pages, constructing with recycled materials, play‐dough creations, and decoupage. A set routine was followed in each session and task chunking was demonstrated via a visual, picture‐based routine and a color‐coded analogue clock to support executive functioning, emotional regulation, and behavior.

Participants had five minutes to engage in free regulatory and autonomous play before washing their hands, eating a healthy snack served with fruit juice, and participating in the greeting circle. Step‐by‐step instructions were provided along with a visual demonstration of the final product. Participants were reminded of the group norms and the group incentive behavioral checklist criteria before they had 20 minutes to participate in the art activity. As the groups continued, the content of art activities became more abstract, requiring more emotional regulation, social communication, individual initiative, and collaboration, according to the grading scale outlined in Vona du Toit's levels of creative ability (de Witt 2014).

### 2.7 Incentives

Caregivers received money each week to assist with transport costs, and participants had juice and a healthy snack at the start of each group. At the end of the study, each child received art supplies and educational materials, and caregivers were provided with a diagnostic screening report and recommendations to assist them with further medical and educational intervention.

### 2.8 Data Analysis

Data were analyzed as per the guidance of a biostatistician. Demographic data were described according to descriptive statistics (measures of central tendency and variability). Categorical demographic data were analyzed in frequency or percentage tables where mode scores were reported.

Other categorical variables were summarized using count (percentage) and continuous variables using mean (standard deviation) or median (interquartile range), depending on the distribution. Ordinal and interval data from the COSA and CPA measures were analyzed according to a paired t‐test (parametric statistics). The COPM measure contains ordinal data and could be analyzed with the Wilcoxon signed‐rank test (non‐parametric statistics); however, the biostatistician advised that a paired t‐test would be sufficient for statistical analysis, given similar results on both tests. Consequently, pre‐ and post‐intervention CPA, COPM, and COSA measures were compared using paired t‐tests, with significance set at p<0.05, and Cohen's d was used to determine effect sizes (Lakens 2013). According to Lakens et al., a Cohen's *d* value of 0.8 indicates a large clinical effect size, with medium and small effect sizes being ranked, D. 2013 at *d* = 0.5 and *d* = 0.2, respectively (Lakens 2013). The data analysis methods used are detailed in Table 2.

**TABLE 2:** Data analysis methods.

Objective	Outcomes/Variable	Variable type	Predictors/Comparisons	Analysis/Presented
To report participants’ and caregivers’ demographic factors, including age, diagnosis, household characteristics, income, language, level of education, occupation, place of residence, and sex.	Age, diagnosis, household characteristics, income, language, level of education, occupation, place of residence, and sex.	Sex, language, diagnoses, place of residence, and household characteristics—Nominal data. Number of people living at home—Discrete data. Age—continuous data Income, and level of education—Ordinal data.	N/A	Mean, mode, min, max, range, and standard deviation Frequency/ percentage tables for nominal data.
To determine the limited efficacy of the intervention by comparing pre‐ and post‐intervention scores via the COPM, CPA, and COSA assessments.				
COPM	Level of occupational performance and satisfaction.	Ordinal	Pre‐ and post‐intervention scores	Paired *t*‐test Cohen's *d*
CPA	Level of creative ability and phase of development (therapist‐directed, patient‐directed, and transitional).	Interval	Pre‐ and post‐intervention scores	Paired *t*‐test Cohen's *d*
COSA	POMP (Standard scores).	Interval	Pre‐ and post‐intervention scores	Paired *t*‐test Cohen's *d*

## 3 Results

### 3.1 Potential Clinical Outcomes

#### 3.1.1 Canadian Occupational Performance Measure

On average, there was a larger than two‐point improvement in both COPM subscales from pre‐ to post‐intervention, indicating a clinically significant improvement in participants’ occupational performance (Law et al. 2015). The mean COPM score pre‐test was 3.24 (SD = 1.32), while the post‐test was 5.57 (SD = 1.07). The mean difference between the pre‐test and post‐test scores of 2.33 was statistically significant, *t* (7) = 4.25, *p* = 0.0038. The effect size for the difference between the pre‐test and post‐test was calculated using Cohen's *d*, resulting in a value of 1.5. Moreover, caregivers’ satisfaction with their children's occupational performance improved significantly from pre‐ to post‐test, according to the Cohen's *d* value of 1.356. Therefore, the Cohen's *d* values indicate that the intervention effectively improved caregivers’ ratings of their children's performance, with a large effect size.

**FIGURE 1:**
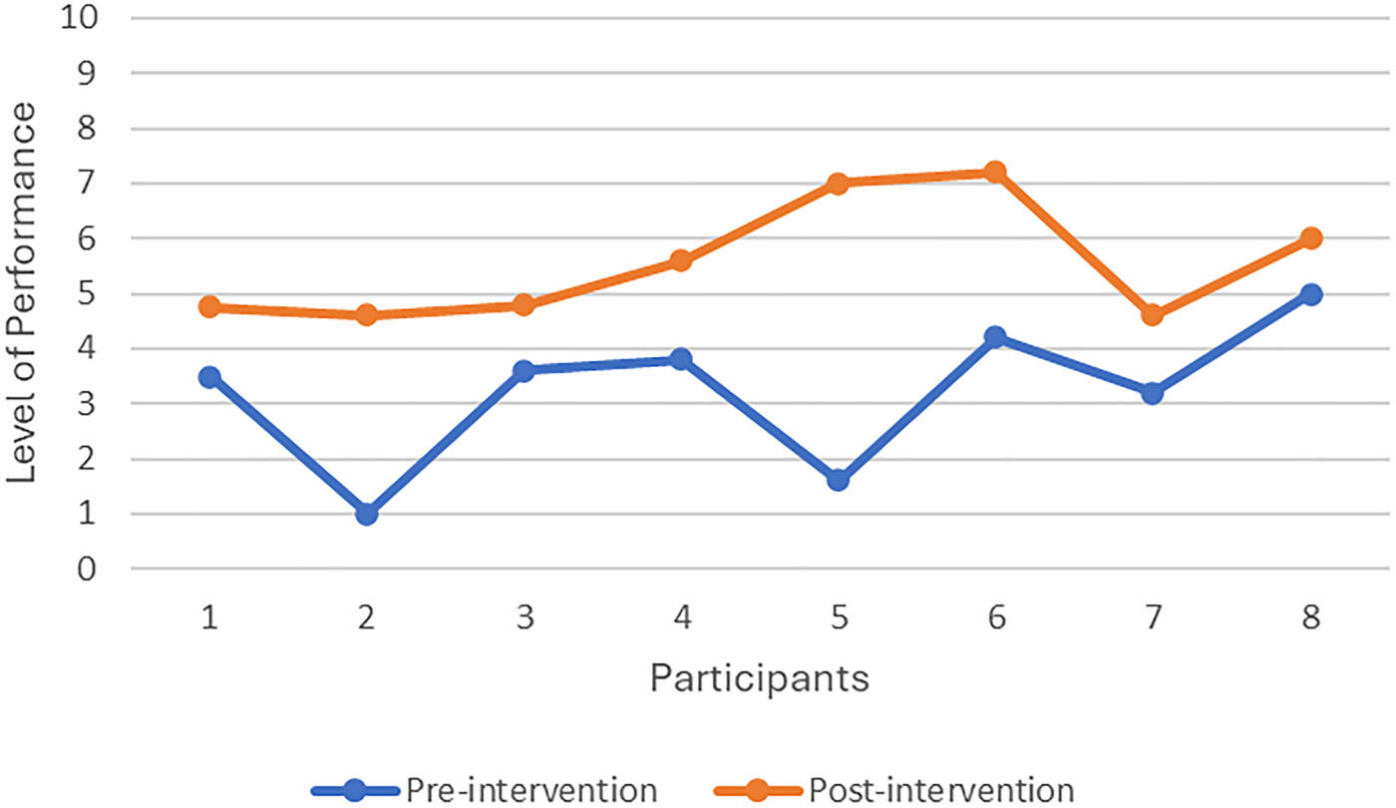
COPM performance ratings.

**FIGURE 2:**
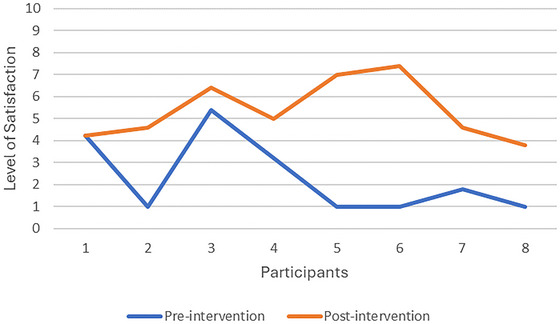
COPM satisfaction ratings.

Figures 1 and 2 illustrate that as participants’ occupational performance improved, so did their caregivers’ satisfaction with their occupational performance in the five occupational performance problem areas identified at the start of the study. Only one caregiver satisfaction score remained unchanged from pre‐ to post‐test, despite an increase in occupational performance.

#### 3.1.2 Creative Participation Assessment

The CPA levels and phases were converted to interval data on a scale of 1–21. The mean CPA at the pre‐test was 11.1 (SD = 2.75), indicating that the average CPA level was passive participation in a participant‐directed phase. At the post‐test, the mean CPA was 13.63 (SD = 2.33), indicating that the average CPA level was the imitative participation level in a participant‐directed phase.

The mean difference between the CPA post‐test and pre‐test was 2.5 (SD = 1.41). Analysis using a two‐sided paired t‐test indicates a significant difference, *t* (7) = 5, *p* = 0.002. This mean difference of 2.5 shows that, on average, participants improved by two to three phases on the CPA. Analysis of the effect of the intervention using Cohen's *d* indicated a large effect size (*d* = 1.76).

#### 3.1.3 Child Occupational Self‐Assessment

On the COSA, the mean difference between the pre‐test and post‐test was 2.37 (SD = 16.05), indicating no significant statistical difference (p = 0.069).

**FIGURE 3:**
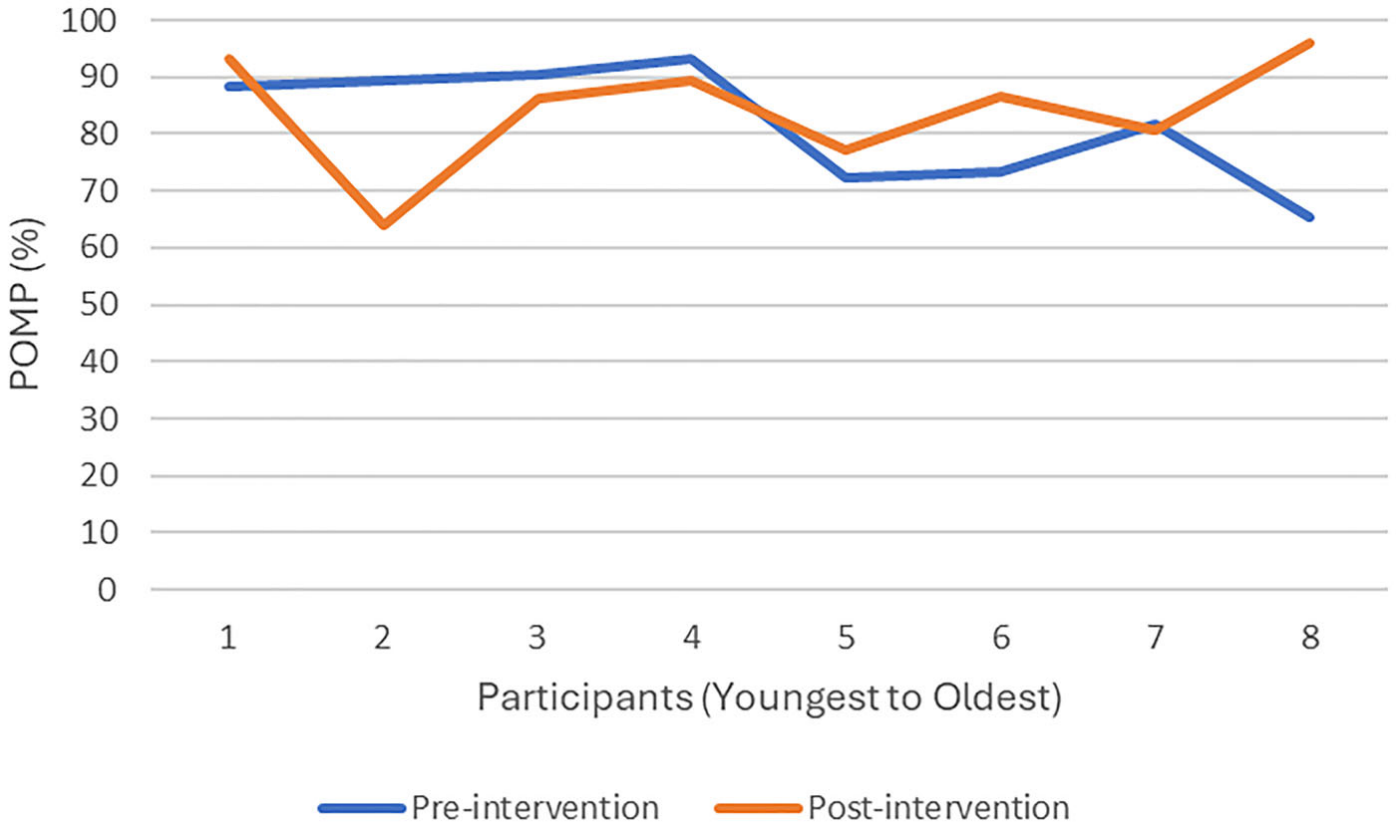
COSA scores (youngest to oldest).

Figure 3 reflects the trend in COSA scores rated by participants according to age. One learner's poor executive and intellectual functioning and the varied translations received from his caregivers during the assessment's administration rendered his COSA score invalid. In Figure 3, he is represented as participant number one. Participants one to five on Figure 3 ranged from 8 to 9 years old. Therefore, only one of the 9‐year‐old participants improved in his self‐rated occupational competence from pre‐test to post‐test, and the other 8‐ to 9‐year‐old participants decreased in their occupational competence ratings. In contrast, the older children's self‐rated occupational performance increased, except for one participant, whose COSA score remained constant from pre‐ to post‐test.

The statistical analyses used for each pre‐ and post‐intervention assessment outcomes are indicated in the paired *t*‐test results and Cohen's *d* evaluations outlined in Table 3.

**TABLE 3:** Assessment statistical analyses.

CPA, COPM, and COSA Paired t‐test Results					
Variable	Observations	Mean	Standard error	Standard deviation	Confidence Interval (95%)
CPA Pre‐intervention	8	11.13	.	2.75	0.97
CPA post‐intervention	8	13.63	.	2.33	0.82
Difference	8	2.5	. 50	1.41	1.32 – 3.68
H0: mean (diff) = 0, *t* (7) = 5, *p* = 0.002 Cohen's *d*: 2.5/ 1.41 = 1.76 (large effect size)					
COPM Performance Pre‐intervention	8	3.24	. 47	1.32	2.13 – 4.34
COPM Performance Post‐intervention	8	5.57	. 38	1.07	4.67 – 6.46
Difference	8	2.33	. 55	1.55	3.63 – 1.03
H0: mean (diff) = 0, Degrees of freedom = 7, *p* = 0.0038 Cohen's *d*: 2.33/1.55 = 1.5 (large effect size)					
COPM Satisfaction Pre‐intervention	8	2.33	. 62	1.74	. 88 – 3.79
COPM Satisfaction Post‐intervention	8	5.38	. 48	1.36	4.25 – 6.52
Difference	8	3.05	. 80	2.25	4.93 – 1.17
H0: mean (diff) = 0, Degrees of freedom = 7, *p* = 0.0064 Cohen's *d*: 3.05/2.25 = 1.356 (large effect size)					

#### 3.1.4 Mitigating Attrition

Overall, the study had an attendance rate of 90.63% and an assessment completion rate of 96.88%. This indicates a low attrition rate. Moreover, caregivers rated the intervention as in‐demand, practical, and acceptable on 5‐point Likert scales from pre‐ to post‐intervention.

### 3.2 Demographic Data

All the participants were Afrikaans‐speaking, except for one learner who spoke isiXhosa and Sotho. The group consisted of five males and three females, with an average age of 9.38 years. Most were in Grades 2 to 3. The oldest participant was in Grade 6, and one of the children had no formal schooling. Most participants had not attended preschool, given the cost of preschooling and the only recent adoption of the Basic Education Laws Amendment (BELA) Act in September 2024, which makes the attendance of Grade R compulsory (Zero Drop‐Out Campaign 2024). Half of the group had a suspected SLD (such as dyslexia), and AD/HD and ID were the second most common suspected conditions, based on the initial screenings. Comorbid AD/HD, ID, and challenges with verbal communication skills were also detected. The frequency and percentage of these suspected diagnoses are listed in Table 4. None of the participants had ever had formal diagnostic assessments, and the psychologist's screenings provided caregivers with the first indication of their children's learning differences.

**TABLE 4:** Participants’ diagnoses.

Participant diagnoses	SLD	ID	AD/HD	Language disorder	Comorbidities
Frequency	4	3	3	1	5
Percentage (%)	50	37.5	37.5	12.5	62.5

Half of the participants’ families received government‐funded childcare grants, and three caregivers reported frequent food insecurity within their households. Two families had no electricity, and one had no running water. Five lived in formal housing, while the rest lived in makeshift settlements. The majority of the children lived with five or more people. 71.43% of caregivers were unemployed, except for four caregivers who were seasonal farm workers. Several participants were in the care of older relatives, and only two out of 14 recorded caregivers had completed high school. Ten of the caregivers were female, and four were male, with 85.71% speaking Afrikaans. Some caregivers reported not being in good health, with HIV, hypertension, diabetes, asthma, hypercholesterolemia, back pain, and a heart condition being listed; yet, no diagnosed learning or mental health challenges were reported. Familiarity between participants’ caregivers before the study was unknown. Demographic data related to caregivers can be found in Table 5.

**TABLE 5:** Caregivers' age, education, income, and number of residents.

	N	Min, Max	Most	Mean	SD
Caregiver age	14	Min: 30, Max: 64	.	44	13.23
Caregiver education	14	Min: Grade 1, Max: Grade 12	Grade 11	Grade 9	.
Caregiver income	14	Min: No income, Max: < R6000 p/m	<R2000 p/m	< R3000 p/m	.
Number of residents	46	Min: 3, Max: 8 (per household)	.	6 (per household)	1.71

## 4 Discussion

This research study aimed to determine the potential outcomes of eight weekly 45‐minute occupational therapy visual art groups for eight children (aged 8 to 12 years) with learning differences in a rural, low‐resource setting in the Western Cape, South Africa. Data analysis on the COPM and CPA indicated that the intervention was effective in improving the creative ability of children, as several elements in the study and intervention design predisposed the program to success.

The incorporation of caregivers’ perspectives and the opportunity for them to observe the groups if they wished may have positively influenced the intervention's appeal. The COPM proved to be a valuable tool in building rapport with caregivers from pre‐ to post‐intervention, as it allowed them to prioritize occupational performance concerns according to their perspective, collaboratively set therapeutic goals for their children, and observe their growth throughout the groups.

Another group of researchers experienced the beneficial effect of working alongside caregivers as they sought to use community‐based activities to support the functioning of young people with physical disabilities (Law et al. 2015). Aiding participants in identifying their own occupational performance concerns and setting goals with the therapist according to the COPM appeared to encourage their participation in the intervention, thereby enhancing the effectiveness of the program (Law et al. 2015). Keller et al. also emphasized the importance of a collaborative approach to the meaningful evaluation of childhood development, as various ecocultural factors such as local values and practices inform what caregivers view as healthy, desirable, and normal in childhood (2005). Future research evaluations should therefore consider using more participatory methods to ensure the use of culturally appropriate outcome measures that can help foster community participation (Mthembu 2021).

From this research, it is clear that South African healthcare workers ought to view themselves as change agents and not just professionals, as interventions cannot be successfully executed without flexible, creative, and contextually relevant approaches (Case‐Smith and Clifford O'Brien 2014). Unpredictable weather, flooding, last‐minute school roster changes, and challenges such as the lack of transport and telecommunications highlighted that without the holistic lens of community‐based and client‐centered practice, external barriers will continue to hinder equal participation in developmentally appropriate activities in rural, low‐resource contexts (Dhillon et al. 2010; Law et al. 1990; Townsend and Wilcock 2004). This approach is essential given the inequity in occupational therapy service delivery in Africa (Plastow et al. 2023). To mitigate these factors, the research team provided transportation money to participants’ caregivers at the end of each session and initiated appointment scheduling and transport to assessment sessions as needed.

In consideration of the ecocultural elements and health inequities that shaped the research context, the data highlighted the low socioeconomic background of participants, as most caregivers were unemployed (71.43%), and half were living on childcare grants, amounting to R530 ($29.50) per child per month. Consequently, not all of the participants’ basic needs were being met (Macpherson et al. 2016; Witthaus 2015). Unfortunately, all except two of the participants were in public schooling in overcrowded classrooms, making individual support a scarcity. Moreover, a lack of nourishment, low maternal education levels, limited early learning stimulation, and other risk factors such as a lack of accommodative schooling, diagnostic assessments, and specialized intervention highlighted that without support, children from this context are unlikely to realize their creative capacity—the creative potential one has and could reach within ideal circumstances (Burger et al. 2020; de Witt 2014; McCoy et al. 2016; Zablotsky et al. 2019). Therefore, although the sample size in this study was small, the context of participants is representative of the resource constraints and challenges prevalent in the community (Truter et al. 2025), and the Likert‐scale measurements for demand, acceptability, and practicality were beneficial in gauging caregivers’ perspectives on the suitability of the intervention provided.

Beyond the cultural and caregiver considerations that assisted in maintaining a low attrition rate, it is suspected that other factors enabled *Create2Grow* to be effective in improving creative ability. The program offered an alternative means for participants to experience exploration, competence, achievement, autonomy, and relatedness via a process‐oriented approach (Case‐Smith and Clifford O'Brien 2014; Casteleijn et al. 2019; Ryan and Deci 2017). Moreover, the use of fun and age‐appropriate creative activities that elicit just‐right challenges within a structured and nurturing social environment appears to have developed the underlying enablers needed for participation in everyday tasks. This, in turn, effectively enhanced participants’ overall volition and sense of industry (Casteleijn et al. 2019; Law et al. 1990). This was seen in children's increased participation skills during the groups and improved functioning in self‐care, social communication, academic performance, and independence within household tasks, according to the CPA and COPM measures.

This approach was also found to be effective in a study where special school visual arts educators found the processes related to participating in artmaking (e.g., free expression, achievement, and the development of self‐confidence) to be more useful in enhancing the general development of children with ID than art lessons with skills and knowledge‐based outcomes (de Witt 2014; Tam 2016). This suggests that the quality and approach of interventions, particularly those that are process‐driven and client‐centered, may be more effective than interventions of a longer duration lacking these components (Conolly et al. 2025).

Limitations to this study included a small sample group, the lack of a control/comparison group, and concerns regarding the appropriateness of the COSA for the target population, given several respondents’ fatigue and limited comprehension, self‐insight, and attention during the assessment. The relevance of the COSA pictorial scales for children who have not attended school should be considered in future studies.

## 5 Conclusion

The *Create2Grow* pilot study generated a large statistical and clinical effect, according to Cohen's *d* evaluations, in improving the creative ability of participants from pre‐ to post‐test, according to the CPA and COPM ratings. The participants showed trends of improvement in the COSA, but the outcome was not statistically significant (*p* = 0.6889). This is thought to be the result of some children's developing self‐insight and fluctuating attention spans, which made self‐evaluation challenging. Moreover, the groups were found to be in demand, practical, and acceptable according to caregivers. A 90.63% attendance rate and the completion of all but one pre‐intervention CPA rating, given a participant's absence in the first group, indicated high protocol compliance and successful implementation. Overall, this study indicates that the *Create2Grow* program can develop the creative ability of children with learning differences in rural, low‐resource settings, to the extent that it positively impacts their everyday participation and performance in developmentally‐relevant activities. Therefore, *Create2Grow* could be prioritized for further expansion and effectiveness testing.

### 5.1 Recommendations

- It is recommended that clinicians, therapy assistants, and community workers be trained in *Create2Grow* and similar, resource‐conscious, and group‐based approaches to increase access to developmental support services in rural, low‐resource settings in Africa.
- Clustering children into narrower age brackets, such as ages 10 to 12 years old, may improve social cohesion and psychosocial development outcomes amongst participants.
- The *Create2Grow* groups could be lengthened to 10 weeks to reinforce a sense of belonging, relatedness, and achievement, especially for younger participants.
- Conducting research in rural, low‐resource settings requires flexibility and resilience. Challenges such as a lack of telecommunications and transport and certain cultural beliefs that stigmatize learning differences need to be taken into account. Therefore, it is recommended that future researchers seeking to conduct similar studies remain adaptable and kind, going out of their way to coordinate logistics on behalf of participants (such as contacting caregivers directly instead of waiting for them to make contact and offering participants from surrounding farms transport) and gently challenge unhelpful beliefs about neurodiversity and participants’ performance potential.

## Author Contributions

N. B. C. was responsible for project administration, conceptualization, investigation, funding acquisition, methodology, validation, visualization, software management, formal analysis, resource management, data curation, writing the original draft, and reviewing and editing the manuscript. M. H. and N. A. P. provided supervision for all stages of the research and assisted with reviewing and editing the manuscript. D. M. assisted in research investigation, including participant recruitment, DSM‐V screening, and the co‐facilitation of intervention groups. J. vd. W. was responsible for administering the pre‐ and post‐intervention assessments and assisting in reviewing and editing the original draft.

## Funding

The Neurodiversity Foundation, specifically Mr. Ben Truter, is acknowledged for its financial assistance during this research.

## Ethics Statement

Ethical approval was obtained from the Health Research Ethics Committee affiliated with Stellenbosch University before commencing the pilot study. **Project ID**: 24905. **Ethics Reference Number**: S23/01/013.

## Consent

Consent was obtained from primary caregivers, and verbal assent was obtained from research participants before the pilot study.

## Conflicts of Interest

The authors declare no conflicts of interest.

## Data Availability

The data that support the findings of this study are available from the corresponding author upon reasonable request.
